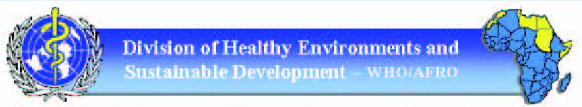# EHPnet: WHO/AFRO Division of Healthy Environments and Sustainable Development

**Published:** 2005-08

**Authors:** Erin E. Dooley

The people of Africa are besieged by a wide range of diseases that are hard to eradicate because of widespread lack of sanitation and medical facilities. A number of factors—including poverty, lack of technology, undeveloped infrastructure, and political conflict—mean that the vast mineral, water, and forest resources of the continent for the most part are not sustainably managed, leaving ecosystems degraded, biodiversity severely affected, and human health at risk. The World Health Organization Regional Office for Africa (WHO/AFRO) has implemented a Division of Healthy Environments and Sustainable Development to identify, control, and prevent environmental conditions that adversely impact human health in the context of sustainable development. The division has set up a website at **http://www.afro.who.int/des/index.html** to educate the public about its activities.

The homepage features overviews of the division’s 12 focus areas: coordination of macroeconomics and health; environmental health policy; environment and promotion of health; environmental risk assessment; food safety; health action in times of crisis; health in sustainable development; long-term health; occupational health; poverty and ill health; protection of the human environment; and water, sanitation, and health. Each area has its own subpage with links to relevant publications and other related resources.

Work in the environmental health policy arena concentrates on assisting countries in developing and implementing environmental health policies, and in building and strengthening nations’ capacity to offer sound environmental health services. Falling under the umbrella of environmental risk assessment are initiatives to improve and promote drinking water quality, chemical safety, environmental health impact assessments and mapping, sustainable management of biomedical wastes, and radiation safety.

One of the more in-depth sections of the website covers food safety. Contained here are fact sheets for health care workers on topics such as genetically modified foods, mycotoxins, informal food trading, groups at high risk for foodborne illness, and hand-washing to prevent disease, among others. This section also includes profiles of 28 African countries with statistics on population, food production and consumption, food-related legislation, and other related topics. Other links go to the WHO’s main food safety pages and a photo gallery depicting the many problems encountered by Africans in obtaining safe food.

The occupational health section provides a link to the WHO/International Labour Organization Joint Effort on Occupational Health and Safety in Africa site, available in English, French, Portuguese, and Arabic. This effort aims to bring occupational safety and health professionals from throughout Africa together in a collaborative network. Also available is contact information for the two occupational health training centers on the continent and links to WHO publications on the subject. The protection of human environment section has profiles for the 46 African countries under the jurisdiction of the WHO/AFRO. The profiles list details about the environmental health laws in each country, and in some instances about plans for developing and implementing new policies.

The water, sanitation, and health section has information on the Africa 2000 Initiative. Launched in 1994 by the health ministries of the 46 WHO/AFRO nations, this initiative seeks to expand water and sanitation services throughout Africa. This section also has information on the Participatory Hygiene and Sanitation Transformation Initiative, a program developed by the WHO and other partners to promote community management of water and sanitation resources.

## Figures and Tables

**Figure:**